# Improving paediatric immunisation coverage rates: a position paper of the European Academy of Paediatrics

**DOI:** 10.1016/j.lanepe.2026.101851

**Published:** 2026-09-04

**Authors:** Hans Jürgen Dornbusch, Zoi Dorothea Pana, Stefano del Torso, Adamos Hadjipanayis, Joe Brierley, Nora Karara, Ann De Guchtenaere, Mark Buttigieg, Zachi Grossman, Iren Kantor, Luigi Titomanlio, Corinne Wyder, Sophie Jullien, Brett Janson Craig, Roberta Pastore, Oleg Benes, Ulrich Heininger, Berthold Koletzko

**Affiliations:** a Institute for Hygiene, Microbiology and Environmental Medicine, Medical University of Graz, Graz, Austria; b Department of Basic and Clinical Sciences, Medical School, University of Nicosia, Nicosia, Cyprus; c ChildCare WorldWide ODV, Padova, Italy; d Medical School, European University Cyprus, Nicosia, Cyprus; e Paediatric Bioethics Centre & Paediatric Intensive Care, Great Ormond Street Hospital, London, UK; f Universitätsklinikum Ruppin-Brandenburg, Neuruppin, Germany; g Department of Paediatrics and Internal Medicine, Ghent University, Ghent, Belgium; h Department of Child and Adolescent Health, Gozo General Hospital, Malta; i Sheldon School of Medicine, Ariel University, Ariel, Israel; j Department of Paediatrics, Szabolcs-Szatmar-Bereg County Hospital, Nyiregyhaza, Hungary; k Pediatric Emergency Department, Robert Debré University Hospital, Assistance Publique-Hôpitaux de Paris, Paris Cité University, Paris, France; l Kinder- und Jugendmedizin KurWerk AG and Department of Medicine, University of Bern and Fribourg, Bern, Switzerland; m World Health Organization Regional Office for Europe, Copenhagen, Denmark; n Ulrich Heininger, University of Basel Children‘s Hospital and Faculty of Medicine, Basel, Switzerland; o LMU University of Munich, Dept. of Paediatrics, Dr. von Hauner Children’s Hospital, LMU University Hospital and German Center for Child and Adolescent Health, site Munich, Germany; p Paediatric and Adolescent Medicine Practice, Graz, Austria; q Maccabi Health Services, Tel Aviv, Israel

**Keywords:** Childhood immunisation, Vaccine hesitancy, Vaccination coverage, Immunisation programmes, Disease surveillance, Immunisation information systems, Digital vaccination records, Public health, Measles

## Abstract

The European Academy of Paediatrics reaffirms its strong support for comprehensive childhood immunisation programmes. At a time when vaccine hesitancy, policy instability, and funding gaps threaten established achievements, public health policy must remain firmly grounded in scientific evidence, transparency, and ethical responsibility. Based on established evidence and current post-pandemic data, we urgently endorse Europe-wide harmonised surveillance systems for vaccine-preventable diseases, interoperable Immunisation Information Systems (IIS), and a Pan-European standard for digital vaccination records or routine measurement of vaccine confidence and access barriers; and equity-focused catch-up immunisation for under-immunised groups. Investment is also needed in training and tools that equip paediatricians and all other healthcare professionals involved in immunisation to build confidence in vaccination. Coordinated, multilingual public information campaigns should proactively counter misinformation and visibly align political decisions with scientific evidence.

## Short introduction

Decades of accumulated scientific evidence demonstrate that immunisation programmes substantially reduce morbidity and mortality from infectious diseases and protect both individuals and communities. In this context, the European Academy of Paediatrics (EAP) has unequivocally affirmed its position in support of scientifically evaluated childhood immunisation, emphasizing vaccination as a fundamental pillar of child health and public health policy.^1^ However, despite this strong evidence base and policy consensus, persistent under-vaccination and emerging immunity gaps–driven by inadequate training and communication, vaccine confidence concerns based on misinformation, inequitable access, fragmented documentation, and other system-level barriers–are increasingly threatening these public health gains and have led to the re-emergence of vaccine-preventable diseases across Europe, particularly during and after COVID-19 pandemic.^2^ The present concern is therefore not vaccine efficacy but reach: COVID-19 related disruptions amplified existing inequities, and recent WHO/UNICEF data show that childhood vaccination coverage in Europe and Central Asia remained below pre-pandemic levels in 2024, with wide country variation.^3^^,^^4^

## The multiple benefits of vaccination

Vaccination remains one of the most effective and impactful preventive interventions in paediatric medicine. Modelling of 50 years of the Expanded Programme on Immunization shows a major contribution of vaccination to improved survival and health^5^ Ongoing advances in vaccinology continue to demonstrate the safety and effectiveness of the vaccines recommended for use in children. For example, recent evaluations of meningococcal B vaccines show how careful development, rigorous surveillance, effective communication, and real-world data can result in highly effective and safe vaccines that substantially reduce severe childhood disease.^5^^,^^6^; HPV vaccination has been associated with a substantial reduction in invasive cervical cancer risk^7^; and rotavirus vaccination has been recognised as highly effective tool to reduce severe gastroenteritis and hospitalisation in children.^8^^,^^9^ Such successes reinforce confidence in immunisation as a cornerstone of paediatric preventive medicine, but only when communicated transparently, consistently, and in language that parents and communities can understand. In a recent policy statement, the American Academy of Pediatrics (AAP) points out that vaccinations are among the most effective preventive measures and avert not only health risks but also economic damage.^10^ For the ten-year period from 1994 to 2003, it is estimated that 500 million cases of vaccine-preventable diseases, 32 million hospitalisations and 1.1 million deaths were avoided, saving $540 billion in direct costs and $2.7 trillion in indirect costs during that period in the USA. Vaccination as a strict requirement for admission to day care facilities or school in all 50 US states contributes to individual and community protection of individual classes or facilities including the staff and in the family environment. Recent studies confirm effectiveness of vaccination programmes in reducing economic and disease burden also in the European setting.^11^, ^12^, ^13^

Vaccines also play an important role in curbing antimicrobial resistance by preventing infections that would otherwise require antibiotic treatment, thereby reducing the emergence and transmission of resistant pathogens and lowering overall antibiotic use. These effects have been recognised in policy frameworks by the WHO.^14^

## Current challenges and consequences

Under-vaccination can be framed as the result of the two interacting barriers: access and acceptance. Access barriers include missed opportunities for vaccination during routine health visits; rural or underserved settings where long travel distances, transportation costs and logistical constraints impede service use; weak follow-up and reminder/recall systems; and limited timeliness of series completion. Individual and household-level characteristics, including socioeconomic position, maternal education and health literacy, influence both the ability to navigate services and engagement with vaccination information. Marginalised and mobile groups–including migrants, refugees and other displaced persons–face additional structural and administrative barriers, fragmented or interrupted care, and challenges linked to mobility, legal status and unfamiliarity with health systems. Conflict and other service interruptions can further widen existing inequities in coverage and timeliness.^15^^,^^16^

Acceptance barriers include concerns about vaccine safety or effectiveness, distrust, social norms, misinformation, and low perceived disease risk when vaccine-preventable diseases have become less visible. Socio-demographic situation and health-literacy factors should not only be considered structural factors, but also individual and social determinants, because they influence both the ability to access services and the interpretation of vaccination information.^15^^,^^17^^,^^18^

Across Europe, increasing cross-border mobility of families and adolescents makes reliable vaccination records essential, yet vaccination documentation remains fragmented, often non-digital, and non-interoperable, making immunisation gaps difficult to detect when children move between health systems.^19^ This challenge is particularly pronounced among migrants, refugees and other mobile populations, where disrupted care and incomplete records contribute to lower vaccination coverage. Systematic reviews show that migrant and refugee populations in Europe often experience lower vaccination coverage than host populations because of combined access and acceptance barriers, including administrative and legal barriers, language barriers, gaps in continuity of care, limited information, difficulty navigating health systems, and trust-related determinants.^20^^,^^21^ Recent register-based evidence among Ukrainian refugee children in Norway further illustrates how mobility and incomplete records can leave important gaps in documented childhood vaccination.^22^

This evidence directly supports a pan-European digital vaccination-record standard: not a single central database, but an agreed minimum interoperable dataset, mapped to national schedules, protected by data-governance safeguards, and useable in routine care and catch-up vaccination, especially for mobile children and displaced families.^19^, ^20^, ^21^, ^22^

Vaccine confidence has become a dynamic challenge rather than a static attitude. European data show that parental confidence varies between countries and communities, and confidence can be weakened by institutional mistrust, pandemic fatigue, inconsistent policy messaging, and misinformation.^17^^,^^18^^,^^23^^,^^24^ In Europe, misinformation is commonly amplified through social media, messaging applications, politicised narratives, and anecdotal claims of harm; it gains traction when families experience access barriers, receive inconsistent advice, or perceive vaccine-preventable diseases as remote. Effective responses therefore require more than fact sheets: they require social listening, pre-bunking and rapid correction of false claims, multilingual community engagement, trusted clinician and community messengers, and transparent policy decisions visibly aligned with scientific evidence.^23^

Recent global data indicate that progress in childhood immunisation has stalled or reversed in many regions, leaving millions of children under-vaccinated or entirely unvaccinated, especially after the COVID-19 pandemic.^3^ These immunity gaps have immediate and tangible consequences. A comprehensive review in The New England Journal of Medicine has documented a dramatic resurgence of measles, with more than 395,000 confirmed cases worldwide in 2024 and a further sharp increase reported in early 2025. Beyond acute morbidity and mortality, measles is associated with serious complications such as encephalitis, post-infectious immune amnesia and subacute sclerosing panencephalitis, reinforcing that measles is not a benign childhood illness but a potentially devastating disease preventable through vaccination. The same review highlights that widening immunity gaps now directly affect infants and adults, driven by declining vaccine coverage, delayed schedules and underfunding of immunisation programmes. Early infant vaccination in specific contexts, vitamin A supplementation to reduce complications, and novel vaccine delivery platforms such as microneedle patches are complementary measures, but none can substitute for high routine vaccination coverage.^25^

The consequences are already visible in Europe. In 2024 nearly 300,000 pertussis cases were reported in the European Region, more than a three-fold increase from the previous year.^4^ WHO/UNICEF reported that the European Region recorded more than 127,000 measles cases in 2024, double the number in 2023 and the highest since 1997; children under 5 years accounted for more than 40% of cases and 38 deaths were reported.^26^ Earlier EC/WHO Europe and UNICEF statements had linked missed measles vaccination during and after the COVID-19 period to major resurgence of disease, including an up to 60-fold increase in reported measles cases in 2023 compared with 2022.^27^

## Consequences of inaction

Inaction has epidemiological, individual, economic and health-system consequences. Epidemiologically, susceptibility clusters lead to recurrent outbreaks, cross-border transmission, preventable complications and avoidable deaths. At the individual level, people who develop a vaccine-preventable disease may experience acute illness, hospitalisation, long-term sequelae or disability, and, in some cases, death–outcomes that could often have been prevented through timely vaccination. These consequences may also include interrupted education, reduced quality of life, and psychological, caregiving and financial burdens for affected individuals and their families. Economically, outbreaks divert resources from routine care to emergency response, contact tracing, post-exposure prophylaxis, hospital care and catch-up campaigns, while families face school absence, lost work time and anxiety. At health-system level, failure to invest in surveillance, digital records, workforce training and tailored communication perpetuates a reactive cycle: systems detect gaps late, respond under pressure, and then return to the same vulnerabilities. The cost of inaction is therefore not only the return of vaccine-preventable disease, but also the erosion of trust and resilience in preventive child-health systems.^10^, ^11^, ^12^, ^13^, ^14^^,^^25^, ^26^, ^27^

## What should be done

Preventive paediatric care across Europe remains highly heterogeneous, with marked variation in care models, professional roles, and health-system organisation.^19^ Consequently, not all healthcare professionals currently involved in vaccine delivery have received formal education or adequate background training in immunisation. This expertise is essential, because healthcare professionals must increasingly communicate the benefits and risks of vaccination when the burden of diseases such as polio is no longer visible owing to vaccine success, or when infections lead to serious outcomes many years later, such as SSPE, HPV-related cancers and poliomyelitis-related paralysis.^5^^,^^7^^,^^28^

Recommendations must be common in purpose but adaptable in implementation. Centralised and higher-resource systems may be able to expand real-time national Immunisation Information Systems (IIS), automated reminders and direct electronic linkage to primary-care records. More decentralised or resource-constrained systems may need a staged approach, beginning with a minimum core dataset, sentinel or regional pilots, paper-to-digital transition plans, mobile outreach and periodic data-quality audits. The objective is not identical systems in every country, but comparable data, rapid identification of immunity gaps and accountable action.

Operationalising existing tools is essential. The WHO Regional Office for Europe communication training module for health workers should be delivered through pre-service curricula, continuing professional development, train-the-trainer sessions and local simulation or role-play exercises for consultations with hesitant families.^29^ The WHO Pocket book of primary health care for children and adolescents should be embedded into primary-care, school-health and public-health protocols so that key messages, contraindication guidance and myth-addressing content are used consistently.^30^ WHO Behavioural and Social Drivers of Vaccination (BeSD) tools, the ECDC 2025 social and behavioural science toolkit and the Vaccine Barriers Assessment Tool (VBAT) provide structured ways to measure why coverage is low and to design tailored interventions rather than generic campaigns.^23^^,^^31^^,^^32^ Their use should be monitored by immunisation programmes so that these resources do not remain stand-alone documents.

A minimum monitoring framework would strengthen policy relevance and comparability. Countries should track, at least by age, vaccine, subnational area and relevant equity strata where ethically and legally appropriate: MMR1/MMR2, DTP3, polio, HPV, rotavirus and other nationally recommended vaccine coverage; timeliness and missed-dose indicators; zero-dose and under-immunised children; reminder/recall functioning; stock-outs and service availability; outbreak incidence; reasons for under-vaccination; vaccine confidence and access barriers; and the proportion of vaccinators trained in evidence-based communication. Where IIS are available, coverage and missed-dose data should be monitored continuously and reported at least quarterly; social and behavioural driver surveys can be repeated periodically and after outbreaks or major policy changes. Stanberry et al. have similarly proposed that countries should be assessed on their capacity to collect and use social and behavioural-driver data for vaccine uptake, including as a fourth immunisation indicator in the Joint External Evaluation tool.^33^

Because childhood vaccination is delivered by different professionals across Europe, these recommendations apply not only to paediatricians but also to general practitioners, nurses, school-health services, public-health teams, community health workers where used, and programme managers.^19^^,^^34^ Training should therefore cover vaccine science, contraindications, safety communication, motivational interviewing, culturally competent care, outbreak communication, and use of IIS and behavioural tools.

A recent commentary describes how legal, regulatory and political shifts, combined with the spread of misinformation, have weakened long-standing protections for immunisation programmes.^35^ These changes are associated with declining childhood vaccination rates and the re-emergence of vaccine-preventable diseases, illustrating how policy choices that diverge from scientific evidence can rapidly undermine public health gains.

Ethically, children have a fundamental right to scientifically proven, safe healthcare interventions that protect them from avoidable harm. Professional societies and all healthcare providers involved in immunisation should support parents to make informed vaccination decisions based on evidence, empathy and transparent communication. High, equitable access and trust-building should be the first-line policy response.

Mandatory vaccination can be ethically defensible only under strict conditions: when voluntary approaches have failed, disease risk is substantial, access barriers have been addressed, the measure is proportionate, time-limited and legally authorised, medical exemptions and safeguards are clear, and transparent monitoring protects public trust.^36^

Empathetic, patient-centred communication remains essential; personal narratives of preventable complications may help illustrate the consequences of inaction, but they should support, not replace, transparent scientific evidence and careful benefit-risk communication.^37^

Within this context, the WHO Regional Office for Europe underscores the importance of identifying the drivers of under-vaccination and addressing them by building confidence and improving equitable access to vaccines within and between countries. These priorities are reflected in the WHO/UNICEF Strategy for child and adolescent health and well-being, recently adopted by all 53 Member States, which identifies vaccination as a key concern for the Region. As part of the agreed actions, Member States committed to promote the benefits of vaccination and address vaccine hesitancy, reinforcing the central role of immunisation in safeguarding child and adolescent health and strengthening the resilience of health systems.^38^

## The European Academy of Paediatrics strongly supports the following priority actions (fact box)

The EAP contribution is to translate established evidence on vaccine benefits and emerging evidence on post-pandemic immunity gaps, mobility, misinformation and implementation barriers into the following prioritised, measurable framework:

• System-level surveillance and IIS: strengthen Europe-wide harmonised surveillance for vaccine-preventable diseases and implement or expand national or interoperable IIS to enable real-time or at least quarterly coverage monitoring, detection of missed doses, automated reminders/recalls and rapid outbreak response, with data-protection safeguards.
• Digital vaccination records: establish a pan-European standard for a digital vaccination card or record, linked to national systems, multilingual where feasible, and based on a minimum interoperable dataset that can support cross-border continuity of care and catch-up vaccination.
• Measurement of acceptance and access barriers: incorporate BeSD, ECDC social and behavioural science tools, and/or VBAT into routine programme assessments to measure confidence, social norms, practical barriers, trust, access, and reasons for non-vaccination, and use the results to design and evaluate tailored interventions.
• Equity-focused catch-up: identify immunity gaps by age, geography, mobility and socioeconomic risk where data governance allows; adopt catch-up schedules; screen for missed doses at primary-care, emergency, school-entry and adolescent contacts; and fund outreach to groups at high risk of being missed, including migrants, refugees, hard to reach ethnic or religious communities, underserved urban districts and remote rural areas.
• Provider-level training: ensure that paediatricians, general practitioners, nurses, school-health professionals, public-health vaccinators and other staff involved in immunisation receive structured training in vaccinology, safety communication, motivational interviewing, use of IIS, and management of misinformation; monitor training coverage as a programme indicator.
• Communication and misinformation response: develop coordinated, multilingual communication strategies using trusted clinicians and community messengers, social listening, pre-bunking, rapid correction of false claims, and transparent explanation of policy decisions, including uncertainty and safety monitoring.
• Governance, financing and accountability: protect immunisation budgets, support national immunisation technical advisory groups and programme managers, publish comparable indicators, evaluate interventions, and ensure that political decisions remain aligned with scientific evidence and child-health rights.

## Conclusions

In alignment with previous statements of the European Academy of Paediatrics and the growing body of global evidence, we strongly reaffirm support for comprehensive childhood immunisation programmes. The established evidence is clear: vaccination prevents disease, disability and death, reduces economic burden, and protects health systems. The emerging post-pandemic evidence is equally clear: immunity gaps, cross-border mobility, misinformation, and under-resourced preventive-care systems are creating clusters of susceptible children and renewed outbreaks across Europe. The EAP therefore calls for a shift from high-level advocacy to measurable implementation: interoperable surveillance and IIS, digital vaccination records, routine measurement of behavioural and access barriers, equity-focused catch-up, trained vaccinators across all relevant professions, and transparent communication aligned with science. Protecting children today safeguards the health, trust, and resilience of future generations.

## Contributors

HJD conceptualised and supervised the project, wrote the first draft and finalised the manuscript for submission.

ZDP, SJ, BJC, UH: contributed to conceptualisation of the project, writing of the first draft and finalisation of the project.

SdT, AH, JB, NK, BK: added considerable amendments to the text, critically reviewed the first draft and revised versions of the manuscript.

ADG, RP, OB, ZG, IK, LT, MB, CW: critically reviewed the first draft and revised versions of the manuscript.

## Consent for publication

All authors agree on the current version of the manuscript and give consent for publication.

## Declaration of interests

HJD serves as chair for the European Academy of Paediatrics Immunisation Advisory Group. He has received refunds for product independent lectures from Astra-Zeneca, GSK, MSD and Pfizer.

ZDP is involved on a non-funded basis in a global project (PED IR) supported through a Pfizer Global Grant and in an EU-funded IHI (Innovative Health Initiative) project (READI).

JB serves as chair for the European Academy of Paediatrics Ethics Advisory Group.

UH served as member of the STIKO (Standing Committee on Immunization Practices, NITAG, Germany) from 2019 to 2024. He is a member of the “Global Pertussis Initiative” which receives unrestricted educational grants from Sanofi, has served or serves on data monitoring boards for Seqirus, Novartis, LG Chemics, Takeda, Bionet Asia and the Coalition for Epidemic Preparedness Innovations (CEPI) and has received product independent lecture fees from Bavarian Nordic, Galenicare, GSK, Infectopharm, Merck, MSD, Moderna, Pfizer, Sanofi.

BK serves as the President of the European Academy of Paediatrics which has received funding support for medical education activities from Sanofi and Pfizer.

SdT, AH, NK, ADG, SJ, RP, OB, BJC, ZG, IK, LT, MB and CW declare no competing interests.
